# Preventive Effects of Bee Venom Derived Phospholipase A_2_ on Oxaliplatin-Induced Neuropathic Pain in Mice

**DOI:** 10.3390/toxins8010027

**Published:** 2016-01-19

**Authors:** Dongxing Li, Woojin Kim, Dasom Shin, Yongjae Jung, Hyunsu Bae, Sun Kwang Kim

**Affiliations:** 1 Department of Physiology, College of Korean Medicine, Kyung Hee University, 26 Kyungheedae-ro, Dongdamoon-gu, Seoul 130-701, Korea; leedongxing@naver.com (D.L.); thasnow@gmail.com (W.K.); ssd060@naver.com (D.S.); hbae@khu.ac.kr (H.B.); 2 Department of East-West Medicine, Graduate School, Kyung Hee University, 26 Kyungheedae-ro, Dongdamoon-gu, Seoul 130-701, Korea; 3 Department of Clinical Korean Medicine, Graduate School, Kyung Hee University, 26 Kyungheedae-ro, Dongdamoon-gu, Seoul 130-701, Korea; acuprodigy@gmail.com

**Keywords:** bee venom derived phospholipase A_2_, oxaliplatin, neuropathic pain, regulatory T cells, dorsal root ganglia

## Abstract

Oxaliplatin, a chemotherapy drug used to treat colorectal cancer, induces specific sensory neurotoxicity signs that are aggravated by cold and mechanical stimuli. Here we examined the preventive effects of Bee Venom (BV) derived phospholipase A_2_ (bvPLA_2_) on oxaliplatin-induced neuropathic pain in mice and its immunological mechanism. The cold and mechanical allodynia signs were evaluated by acetone and von Frey hair test on the hind paw, respectively. The most significant allodynia signs were observed at three days after an injection of oxaliplatin (6 mg/kg, i.p.) and then decreased gradually to a normal level on days 7–9. The oxaliplatin injection also induced infiltration of macrophages and upregulated levels of the pro-inflammatory cytokine interleukin (IL)-1β in the lumbar dorsal root ganglia (DRG). Daily treatment with bvPLA_2_ (0.2 mg/kg, i.p.) for five consecutive days prior to the oxaliplatin injection markedly inhibited the development of cold and mechanical allodynia, and suppressed infiltration of macrophages and the increase of IL-1β level in the DRG. Such preventive effects of bvPLA_2_ were completely blocked by depleting regulatory T cells (Tregs) with CD25 antibody pre-treatments. These results suggest that bvPLA_2_ may prevent oxaliplatin-induced neuropathic pain by suppressing immune responses in the DRG by Tregs.

## 1. Introduction

Oxaliplatin is a third-generation platinum-based chemotherapy drug that has recently gained significant importance for treating advanced metastatic colorectal cancer [1,2]. Oxaliplatin is also effective against a wide range of other tumors, such as ovarian, breast, and lung cancers [3,4]. Oxaliplatin is structurally similar to cisplatin but contains a 1,2-diaminocyclohexane carrier ligand. This modification enhances its anti-tumor activity and alters the side effect profile from other platinum-based drugs, as it is not nephrologically or hematologically toxic [5,6]. However, oxaliplatin can cause peripheral neuropathy characterized by dysesthesia in the hands and feet, which is a major dose-limiting side effect [7]. A number of studies have suggested that preventing chemotherapy-induced peripheral neuropathy (CIPN) is important [8], as the chemotherapy time schedule is planned in advance and patients can be treated before the administration of chemotherapeutic agents. Various agents, such as intravenous calcium and magnesium [9], vitamin E [10], and glutamine [11] have been suggested to prevent CIPN. However, despite attempts to find an effective preventive treatment, more data regarding efficacy and safety need to be obtained prior to its general use [12], and no well-accepted preventive therapy has been suggested to date [13].

A number of studies indicate that the immune responses after nerve damage contribute as much to the development and maintenance of neuropathic pain as the initial nerve damage itself [14]. Nerve damage stimulates macrophage infiltration [15] and upregulates pro-inflammatory cytokines, such as interleukin (IL)-1β and tumor necrosis factor (TNF)-α [16,17] in the dorsal root ganglia (DRG) of rodents, which produce neuropathic pain hypersensitivity [18]. Also, depleting macrophages immediately after nerve injury was reported to have clinical potential to prevent neuropathic pain [19]. Several studies have reported that the increased infiltration of macrophages into the DRG [20,21] and secretion of various pro-inflammatory cytokines [22,23] contribute to the development of peripheral neuropathy in CIPN animal models.

Regulatory T cells (Tregs) regulate immune homeostasis by sustaining immunological unresponsiveness to self-antigens and by suppressing excessive immune responses harmful to the host [24,25]. Interestingly, a recent study demonstrated that the increase of Tregs by CD28 superagonist (Treg population expander) in nerve injured and experimental autoimmune neuritis affected rats reduces neuropathic pain hypersensitivity and infiltration of macrophages and other immune cells in peripheral nerves and the DRG [26]. In our previous study, we screened a number of medicinal herbs and venoms that had been traditionally used in Korea and found that Bee Venom (BV) has the greatest effect of modulating Tregs [27].

Phospholipase A_2_ (PLA_2_) is a prototypic group III enzyme hydrolyzing fatty acids in membrane phospholipids, and is one of the major active components of BV [28,29]. PLA_2_ can be found in a variety of sources, such as venoms of bees and cobras, and the pancreas of bovines, but it was suggested that PLA_2_ from different sources performs distinct biological roles by activating different target substrates [30]. We demonstrated previously that consecutive pre-treatment of mice with BV-derived PLA_2_ (bvPLA_2_) significantly decreases the hepatotoxicity and nephrotoxicity evoked by acetaminophen and cisplatin, respectively, by modulating Tregs. Upregulation of pro- inflammatory cytokines, such as TNF-α and IL-6, in liver tissue following acetaminophen administration is decreased by pre-treatment with bvPLA_2_, and this anti-inflammatory effect is nullified in Treg-depleted mice [31]. Furthermore, in cisplatin-induced acute kidney injury model mice, bvPLA_2_ treatment significantly reduces levels of macrophages and pro-inflammatory cytokines in the kidney. This effect is also nullified in Treg-depleted mice, suggesting that Tregs play an important role in the anti-inflammatory effect of bvPLA_2_ [32]. Based on these results, we hypothesized that bvPLA_2_ may attenuate the toxicity induced by various chemotherapeutic agents, such as oxaliplatin, by modulating immune responses.

The aim of this study was to examine whether a PLA_2_ pre-treatment prevents oxaliplatin-induced neuropathic pain in mice by suppressing macrophages and pro-inflammatory cytokines in the DRG via Tregs.

## 2. Results

### 2.1. Preventive Effects of bvPLA_2_ on Oxaliplatin-Induced Cold and Mechanical Allodynia

**Figure 1:**
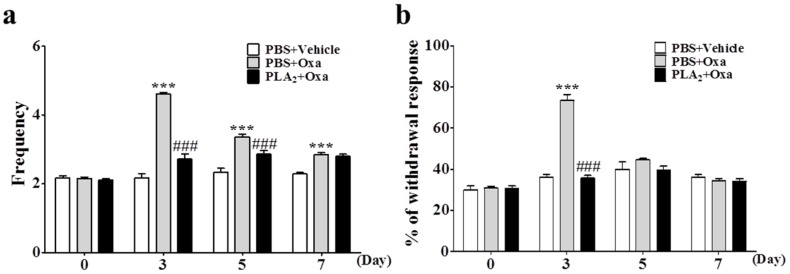
Preventive effects of Bee Venom (BV) derived phospholipase A_2_ (bvPLA_2_) on oxaliplatin-induced cold and mechanical allodynia in mice. (**a**,**b**) The behavioral tests for cold and mechanical allodynia were performed before (day 0) and after administration of oxaliplatin (6 mg/kg, i.p.). The phosphate buffered saline (PBS) + vehicle (5% glucose) (*n* = 8), PBS + Oxaliplatin (*n* = 12), and bvPLA_2_ + Oxaliplatin (*n* = 13) groups received daily injection of PBS or bvPLA_2_ (0.2 mg/kg, i.p.) for five consecutive days before the oxaliplatin or vehicle injection. Results are expressed as mean ± SEM; The data was analyzed with one-way analysis of variance (ANOVA) followed by the Tukey’s multiple comparison test. *** *p* < 0.001, *vs*. PBS + Vehicle; **^###^** *p* < 0.001, *vs*. PBS + Oxaliplatin.

We evaluated the preventive effects of bvPLA_2_ on oxaliplatin-induced cold and mechanical allodynia. Oxaliplatin significantly induced cold and mechanical allodynia compared to that in the vehicle group (5% glucose). Pre-treatment with bvPLA_2_ (0.2 mg/kg/day, i.p.) once a day for five consecutive days significantly reduced cold allodynia from days 3–7 (Figure 1a). The bvPLA_2_ pre-treatment significantly reduced mechanical allodynia on day 3 (Figure 1b). These results suggest that bvPLA_2_ has the potential to prevent oxaliplatin-induced cold and mechanical allodynia.

### 2.2. Inhibition of Macrophages and Pro-Inflammatory Cytokines in the Lumbar DRG by bvPLA_2_ Pre-Treatment

**Figure 2:**
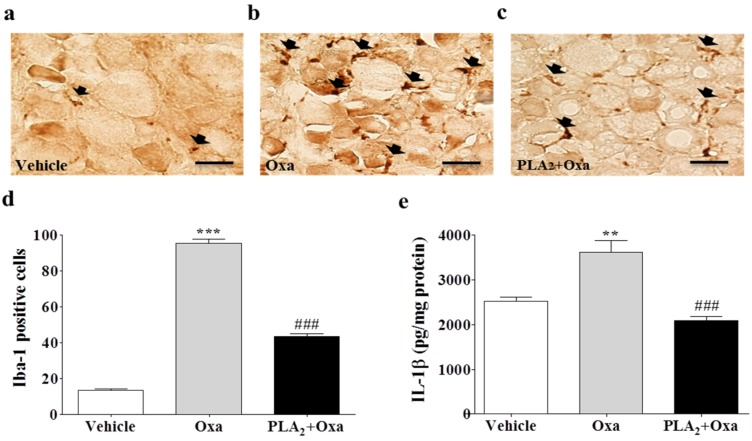
Inhibitory effect of bvPLA_2_ pre-treatment on macrophage and the pro-inflammatory cytokine IL-1β in the lumbar dorsal root ganglia (DRG). (**a**) PBS + vehicle (5% glucose) (*n* = 3); (**b**) PBS + Oxaliplatin (*n* = 4), and (**c**) bvPLA_2_ + Oxaliplatin (*n* = 4) groups received a daily injection of PBS or bvPLA_2_ (0.2 mg/kg, i.p.) for five consecutive days before a single injection of oxaliplatin or vehicle. DRG sections were stained with Iba-1 (macrophage marker) antibody and imaged with a brightfield microscope (original magnification, ×400, scale bar = 200 μm) three days after oxaliplatin administration. Black arrows indicate Iba-1 positive cells. (**d**) Count of macrophages (Iba-1 positive cells) in the lumbar DRG; (**e**) IL-1β concentrations in the lumbar DRG were measured by sandwich ELISA (*n* = 8 mice/group). Results are expressed as mean ± SEM; The data was analyzed with one-way ANOVA followed by the Tukey’s multiple comparison test. ** *p* < 0.01, *** *p* < 0.001, *vs*. PBS + Vehicle; **^###^** *p* < 0.001, *vs*. PBS + Oxaliplatin.

To confirm whether bvPLA_2_ modulates infiltration of macrophages and the increase in the pro-inflammatory cytokines Il-1β and TNF-α in the lumbar DRG, we counted the number of Iba-1 positive macrophages and measured IL-1β and TNF-α concentration after injecting bvPLA_2_ and oxaliplatin. A histological examination (Figure 2a–c) revealed that oxaliplatin significantly increased the number of Iba-1 positive macrophages compared to that in the vehicle group in the lumbar DRG, and the bvPLA_2_ pre-treatment inhibited macrophage infiltration (Figure 2d). In addition, oxaliplatin significantly increased IL-1β level compared with that in the vehicle group, and the bvPLA_2_ pre-treatment inhibited this increase in IL-1β (Figure 2e). The effects of oxaliplatin and bvPLA_2_ on TNF-α levels were similar to those on IL-1β, but no significant difference was found between the groups (Figure S1). These results indicate that bvPLA_2_ inhibits infiltration of macrophages and upregulation of a pro-inflammatory cytokine IL-1β in the lumbar DRG after an oxaliplatin injection.

### 2.3. Effects of bvPLA_2_ Pre-Treatment on Oxaliplatin-Induced Neuropathic Pain in Treg Depleted Mice

**Figure 3:**
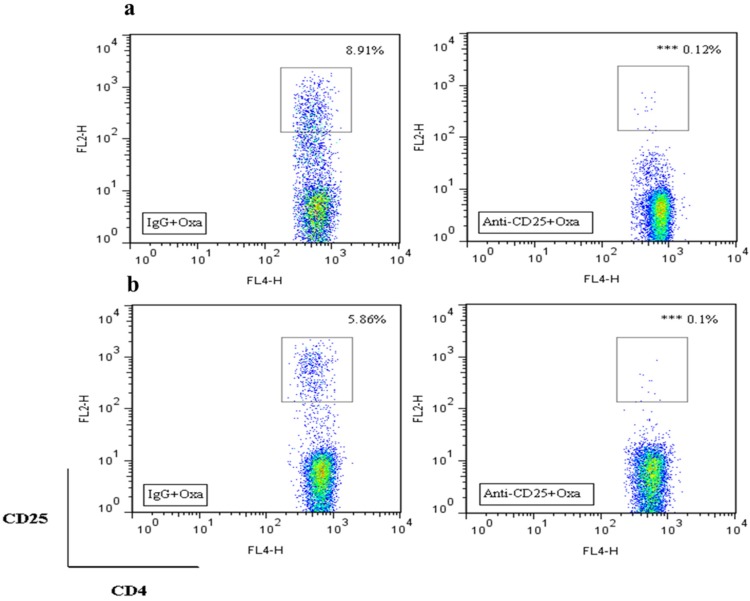
Depletion of CD4^+^CD25^+^ Tregs in Foxp3^EGFP^ mice. (**a**) Confirmation of CD4^+^CD25^+^ Treg depletion in spleen tissue (*n* = 4/group) and (**b**) lymph node tissue (*n* = 4/group). (Right panels) Mice in the anti-CD25 + Oxa group received two injections of 0.1 mg anti-CD25 antibody before the oxaliplatin was administered. (Left panels) Mice in the IgG + Oxa group received IgG injections as a control. Depletion of CD4^+^CD25^+^ Tregs was confirmed by flow cytometry using PE-anti-mouse CD25 and Fluorescein APC-anti CD4 3 days after oxaliplatin administration. *** *p* < 0.001, *vs*. IgG + Oxa, by unpaired *t*-test.

Next, we depleted CD4^+^CD25^+^ Tregs in mice to determine whether the preventive effects of bvPLA_2_ on oxaliplatin-induced neuropathic pain are dependent on Tregs. Anti-CD25 antibody (0.1 mg) was intraperitoneally injected twice, on the day before bvPLA_2_ pre-treatment and on the day before oxaliplatin administration. Depletion of CD4^+^CD25^+^ Tregs was confirmed by flow cytometry of cells from the spleen (Figure 3a) and lymph node (Figure 3b) four days after the final anti-CD25 antibody administration.

**Figure 4:**
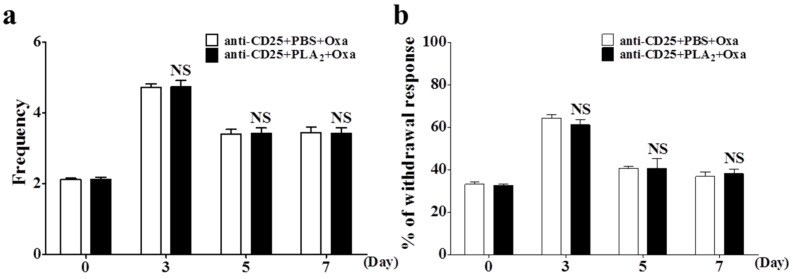
Effects of bvPLA_2_ pre-treatment on oxaliplatin-induced cold and mechanical allodynia in Treg depleted mice. The behavioral tests for cold (**a**) and mechanical (**b**) allodynia were performed before (day 0) and after (days 3, 5, and 7) administration of oxaliplatin (6 mg/kg, i.p.). An anti-CD25 antibody (0.1 mg/mice) was injected twice to deplete Tregs before the bvPLA_2_ and oxaliplatin were administered. anti-CD25 + PLA_2_/PBS + Oxa (*n* = 8/group); Results are expressed as mean ± SEM; NS, no significance (*p* > 0.05), by unpaired *t*-test.

bvPLA_2_ pre-treatment of the CD4^+^CD25^+^ Treg depleted mice had no effect on oxaliplatin-induced cold or mechanical allodynia (Figure 4), which was in contrast to the strong inhibitory actions in naive mice (Figure 1). These results demonstrate that Tregs play a crucial role in the preventive effect of bvPLA_2_ on oxaliplatin-induced neuropathic pain in mice.

### 2.4. Effects of bvPLA_2_ Pre-Treatment on Macrophages and Pro-Inflammatory Cytokines in the DRG of Treg Depleted Mice

**Figure 5:**
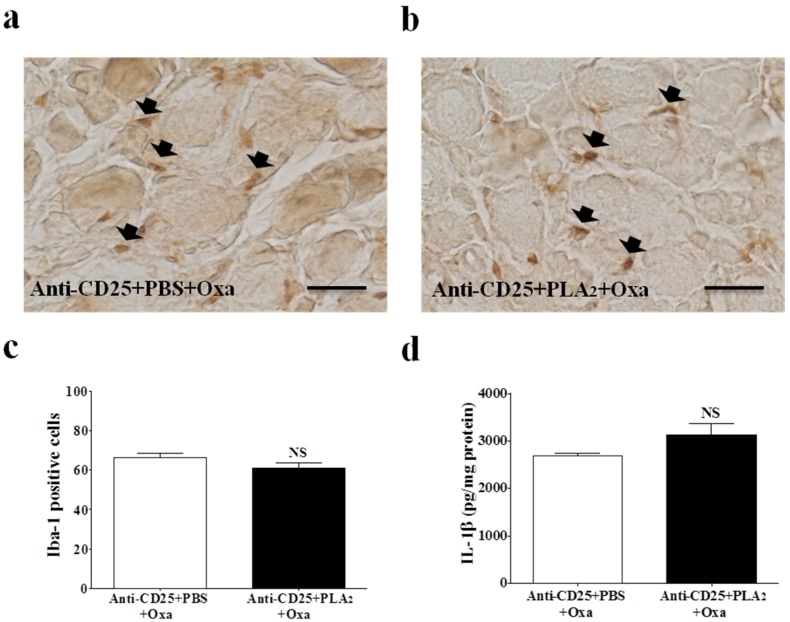
Macrophages and pro-inflammatory cytokine IL-1β in the lumbar DRG of Treg-depleted mice. (**a**) Anti-CD25 + PBS + Oxa and (**b**) Anti-CD25 + bvPLA_2_ + Oxa groups received a daily injection of PBS or bvPLA_2_ (0.2 mg/kg, i.p.) for five days before an oxaliplatin was administered. DRG sections were stained with Iba-1 (macrophage marker) antibody and imaged with a microscope (original magnification, ×400, scale bar = 200 μm) three days after an oxaliplatin administration. Black arrows indicate Iba-1 positive cells; (**c**) Count of macrophages (Iba-1 positive cells) in the lumbar DRG (*n* = 4/group); (**d**) IL-1β concentrations in the DRG of Treg depleted mice were measured by sandwich ELISA (*n* = 8/group). Results are expressed as mean ± SEM. NS, no significance (*p* > 0.05); *vs*. Anti-CD25 + PBS + Oxa, by unpaired *t*-test.

Finally, we evaluated the effects of bvPLA_2_ pre-treatment on macrophage infiltration and IL-1β levels in the lumbar DRG of Treg depleted mice. The histological examination was performed three days after oxaliplatin administration, and the results revealed that the bvPLA_2_ pre-treatment had no effect on macrophage infiltration in the DRG of Treg-depleted mice compared to that of the PBS pre-treatment (Figure 5a–c). Furthermore, no difference was detected in IL-1β levels in the DRG of Treg depleted mice between the PBS and bvPLA_2_ pre-treated groups (Figure 5d). These results indicate that Tregs are required for the anti-inflammatory effect of bvPLA_2_ to decrease macrophage infiltration and the levels of pro-inflammatory cytokines, such as IL-1β, in the lumbar DRG.

## 3. Discussion

Oxaliplatin is a third-generation platinum-based chemotherapeutic drug that is widely used to treat advanced colorectal cancer. It is often used in clinics with other agents, such as 5-flourouracil [33], and capecitabine [34]. Platinum-based chemotherapeutants work via cell phase nonspecific mechanisms to induce cross-linking DNA adducts, and further leading to strand breaks and inhibition of DNA replication [35]. However, oxaliplatin produces side effects, including peripheral neuropathy, diarrhea, and nausea [36], and peripheral neuropathy is recognized as a dose-limiting problem [37]. Although various preventive methods have been suggested, no satisfactory method is available to decrease oxaliplatin-induced peripheral neuropathy. In the present study, we investigated for the first time whether bvPLA_2_ can prevent oxaliplain-induced cold and mechanical allodynia. Our results show that bvPLA_2_ pre-treatment (once daily for five consecutive days) strongly inhibited the development of cold and mechanical allodynia following oxaliplatin administration, suggesting that bvPLA_2_ pre-treatment might be an effective preventive method for oxaliplatin-induced neuropathic pain.

Neuro-inflammation and neuro-immune interactions contribute to the development of neuropathic pain through various immune (e.g., macrophages and T cells) and glial cells (e.g., astrocytes and microglia) [38]. Satellite cells that surround DRG cell bodies proliferate, and neutrophils, macrophages, and T cells are recruited to the DRG in nerve injured animals [39,40,41]. Activated macrophages produce pro-inflammatory cytokines, which are involved in up-regulation of the inflammatory reactions [42]. Abundant evidence indicates that certain pro-inflammatory cytokines, such as IL-1β, TNF-α and IL-6, are involved in the process of pathological pain [43,44,45,46]. Abnormal spontaneous activity from nociceptive neurons can be elicited by topical application of TNF-α to peripheral axons in vivo [47], or to the somata of the DRG neurons in vitro [48]. Localized inflammation in the DRG up-regulates a variety of pro-inflammatory cytokines and induces abnormal sympathetic sprouting in the absence of peripheral nerve injury [49]. IL-1β is released mainly by macrophages and monocytes as well as by non-immune cells, including endothelial cells and fibroblasts, during cell injury, infection, invasion, and inflammation. IL-1β is also expressed in nociceptive DRG neurons [50]. Thus, an immunotherapy that modulates the infiltration of immune cells, such as macrophages, and the upregulation of pro-inflammatory cytokines (e.g., IL-1β and TNF-α) would be a useful preventive treatment for neuropathic pain. In this study, we also found that oxaliplatin significantly increased infiltration of Iba-1 positive macrophages as well as the IL-1β level in the lumbar DRG. Macrophage infiltration and IL-1β upregulation in the DRG following oxaliplatin administration were markedly inhibited by the bvPLA_2_ pre-treatment, suggesting that bvPLA_2_ may exert immunomodulatory actions to prevent oxaliplatin-induced peripheral neuropathy.

Tregs are lymphocytes with immunosuppressive properties that have a crucial role in the maintenance of immune tolerance. Studies using animal models of autoimmune diseases of the nervous system have demonstrated that Tregs inhibit infiltration of macrophages and secretion of pro-inflammatory cytokines in affected regions [51]. Our recent studies demonstrated that pre-treatment of mice with bvPLA_2_ significantly decreases the hepatotoxicity and nephrotoxicity evoked by acetaminophen and cisplatin, respectively, by modulating Tregs [32]. A pain study also reported that the increase in the number of Tregs significantly attenuates neuropathic mechanical allodynia and blocks infiltration of T cells, macrophages and antigen presenting cells in the DRG of nerve injured rats [26]. Therefore, in the present study, we tried to clarify the role of Tregs in bvPLA_2_-induced immunomodulation in oxaliplatin-administered mice. No preventive effect of bvPLA_2_ on oxaliplatin-induced cold and mechanical allodynia was observed in Treg depleted mice. We also found that the bvPLA_2_ pre-treatment had no effect on macrophage infiltration or IL-1β levels in the lumbar DRG of Treg-depleted mice. These results suggest that Tregs are required for bvPLA_2_ to exert preventive actions against neuropathic pain and immune responses in the DRG induced by oxaliplatin administration.

In conclusion, our results demonstrate that bvPLA_2_ pre-treatment effectively attenuated oxaliplatin-induced cold and mechanical allodynia in mice, and that bvPLA_2_ inhibited infiltration of macrophages and decreased IL-1β level in the DRG. Depleting Tregs reversed these preventive effects of bvPLA_2_. Therefore, our results suggest that bvPLA_2_ may have a potent preventive effect on oxaliplatin-induced neuropathic pain through Tregs-mediated suppression of immune responses in the DRG.

## 4. Materials and Methods

### 4.1. Animals

Male C57BL/6 mice (6–8 weeks old) (Charles River Korea, Chungbuk, Korea) were used in most experiments. Foxp3^EGFP^C57BL/6 mice (B6.Cg-Foxp3tm2<EGFP>Tch/J) were purchased from the Jackson Laboratory (Bar Harbor, ME, USA). They were maintained under specific pathogen-free conditions with a 12 h light/dark cycle and air conditioning. The mice had free access to food and water during the experiments. This study was approved by the Kyung Hee University Animal Care and Use Committee (KHUASP(SE)-15-024).

### 4.2. Behavioral Tests

Behavioral tests to examine the different sensory components of neuropathic pain were performed before and after oxaliplatin administration. Before the start of the experiments, the mice were habituated to handling and to all testing procedures for one week. The experimenters were blinded to the oxaliplatin and other treatments.

Cold sensitivity was measured by the acetone test [52]. Mice were placed in a clear plastic box (12 × 8 × 6 cm) with a wire mesh floor and allowed to habituate for 30 min prior to testing. Acetone (10 μL, Reagents Chemical Ltd., Gyonggi-do, Korea) was sprayed onto the plantar skin of each hind paw three times, and the frequencies of licking and shaking of the affected paw were counted for 30 s after the acetone spray.

Mechanical sensitivity was measured by the von Frey hair test [53]. Mice were placed in a clear plastic box (12 × 8 × 6 cm) with a wire mesh floor and allowed to habituate for 30 min before testing. A von Frey filament with a bending force of 0.4 g (Linton Instrumentation, Norfolk, UK) was applied to the mid plantar skin of each hind paw 10 times, with each application held for 3 s [54]. The proportion of withdrawal responses to the von Frey filament applications from both hind paws was quantified.

### 4.3. Oxaliplatin Administration and bvPLA_2_ Treatment

Oxaliplatin (6 mg/kg, Sigma Chemical Co, St. Louis, MO, USA) was dissolved in 5% glucose at a concentration of 2 mg/mL depending on animal weight to ensure intraperitoneal injections of ≤0.5 mL. The vehicle control group received the same volume of 5% glucose solution through the same injection route.

The mice received an intraperitoneal (i.p.) injection of bvPLA_2_ (Sigma) at a concentration of 0.2 mg/kg once daily for five days before oxaliplatin was administered. The control group received an equal volume of PBS. All mice received a single injection of oxaliplatin (6 mg/kg) two days after the last bvPLA_2_ or PBS injection.

### 4.4. Depletion of Tregs

Anti-mouse CD25 rat IgG1 (anti-CD25; clone PC61) antibodies were generated in-house from hybridomas obtained from the ATCC (Manassas, VA, USA). A dose of 0.1 mg of anti-CD25 antibody was injected into Foxp3^EGFP^ mice at the day before bvPLA_2_ treatment and before an oxaliplatin administration. Using PE-anti-mouse CD25 and fluorescein APC-anti-mouse CD4 antibodies, the efficacy of CD4^+^CD25^+^ Treg depletion was confirmed by flow cytometry analysis.

### 4.5. Immunohistochemistry

Immunohistochemical staining was performed to evaluate macrophage infiltration into DRG 3 days after oxaliplatin administration. Briefly, the mice were transcardially perfused with saline and fixed with 4% paraformaldehyde dissolved in 0.1 M phosphate buffer. The L4 and L5 DRGs were removed, post-fixed overnight at 4 °C in buffered 4% paraformaldehyde, and stored in a 30% sucrose solution at 4 °C until they sank. Cryostat sections (12 μm) were made and processed for immunohistochemistry with a primary antibody for Iba-1 (1:500, Wako Pure Chemical Industries, Osaka, Japan). The stained cells were imaged and analyzed under a brightfield microscope (Nikon, Tokyo, Japan). The number of Iba-1-positive cells in each section was calculated by counting the number of positively stained cells in six fields per slide at a magnification of ×400.

### 4.6. Assessment of Cytokines in the DRG by Enzyme-Linked Immunosorbent Assay (ELISA)

IL-1β and TNF-α levels in the DRG were assessed using a quantitative sandwich ELISA kit (BD Biosciences, San Diego, CA, USA for IL-1β and R&D systems, Minneapolis, MN, USA for TNF-α). Frozen DRG tissue was homogenized in a protein extraction solution (PRO-PREP; Intron Biotechnology, Sungnam, Korea) [32]. A 96-well plate was coated overnight at 4 °C with anti-mouse IL-1β and TNF-α monoclonal antibodies (mAbs) in coating buffer. After washing, the wells were blocked with 5% fetal bovine serum (FBS) in PBS and 1% bovine serum albumin (BSA) in PBS for 1 h at 4 °C and room temperature (RT), respectively. The wells were loaded with 100 μL of sample and incubated for 2 h at RT. After washing, secondary peroxidase labeled biotinylated anti-mouse IL-1β and TNF-α mAbs in assay diluents were added for 1 h. Finally, the plates were treated with TMB substrate solution (KPL, San Diego, CA, USA) for 30 min, and the reaction was stopped by adding 50 μL TMB stop solution per well. Optical density was measured at 450 nm in a microplate reader (SOFT max PRO, ver. 3.1 software; Molecular Devices, Sunnyvale, CA, USA, 2008). All results were normalized to the total amount of protein in each sample.

### 4.7. Statistical Analysis

Statistical significance was assessed by one-way analysis of variance (ANOVA) followed by Tukey’s multiple comparison test or by a two-tailed unpaired t test for single comparisons using the Prism 5.01 software (GraphPad Software Inc., La Jolla, CA, USA, 2007). *p* > 0.05 was considered significant.

## Supplementary Materials

The following are available online at www.mdpi.com/2072-6651/8/1/27/s1.
