# Supplementary material for: Preventive Effects of Bee Venom Derived Phospholipase A2 on Oxaliplatin-Induced Neuropathic Pain in Mice
Source: Toxins (Basel). 2016 Jan 19;8(1):27. doi: 10.3390/toxins8010027 (PMC4728549; doi:10.3390/toxins8010027)
Supplement: Supplementary file 1 [file toxins-08-00027-s001.pdf]

Supplementary Materials: Preventive Effects of Bee Venom Derived Phospholipase A2 on Oxaliplatin-Induced Neuropathic Pain in Mice

Dongxing Li, Woojin Kim, Dasom Shin, Yongjae Jung, Hyunsu Bae [1] and Sun Kwang Kim

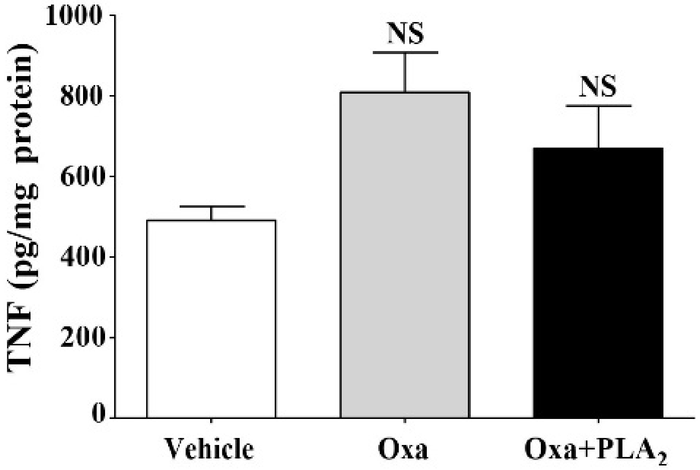

Figure S1. Effect of Bee Venom (BV) derived phospholipase A_2 ($bvPLA_2$) pre-treatment on the pro-inflammatory cytokine, TNF-α in the lumbar dorsal root ganglia (DRG). TNF-α concentrations in the lumbar DRG were measured by sandwich ELISA (n = 4 mice/group). Results are expressed as mean ± SEM; The data was analyzed with one-way analysis of variance (ANOVA) followed by the Tukey's multiple comparison test. NS, no significance ($p > 0.05$).
